# Outcomes of renal replacement therapy in acute kidney injury: factors associated with dialysis dependence and progression to end-stage renal disease – a MarketScan database analysis

**DOI:** 10.1080/0886022X.2025.2504015

**Published:** 2025-05-21

**Authors:** Ahmad Matarneh, Sundus Sardar, Abdelrauof Akkari, Eric Schaefer, Muhammad Abdulbasit, Ronald Miller, Navin Verma, Nasrollah Ghahramani

**Affiliations:** a Department of Nephrology, Pennsylvania State Milton S Hershey Medical Center, USA; b Department of Biostatistics, Penn State College of Medicine, Pennsylvania State Milton S Hershey Medical Center, USA

**Keywords:** Dialysis dependence, acute kidney injury, continuous renal replacement, renal replacement therapy

## Abstract

**Objectives:**

Renal replacement therapy (RRT) is vital for managing acute kidney injury (AKI), with continuous renal replacement therapy (CRRT) and intermittent hemodialysis (iHD) as primary modalities. CRRT is preferred for critically ill patients due to gradual fluid and solute removal, whereas iHD is used for stable patients. Outcomes among AKI patients requiring RRT vary widely, with some recovering kidney function while others progress to end-stage renal disease (ESRD). This study evaluates the risk of dialysis dependence and ESRD within 90 days among AKI patients receiving RRT.

**Methods:**

Retrospective cohort study analyzed inpatient admissions from the MarketScan database (2005–2021) with an AKI diagnosis requiring RRT, identified using ICD-10 codes. Logistic regression compared CRRT and iHD groups, adjusting for age, sex, length of stay, and calendar year.

**Results:**

Compared to iHD, CRRT was associated with 67% lower odds of dialysis dependence at discharge (OR = 0.33; 95% CI: 0.28–0.39) and 80% lower odds at 90 days (OR = 0.20; 95% CI: 0.16–0.27). Patients receiving both iHD and CRRT had higher odds of dialysis dependence at discharge (OR = 1.41; 95% CI: 1.27–1.57) but 46% lower odds at 90 days (OR = 0.54; 95% CI: 0.45–0.64). CRRT also reduced the risk of ESRD within 90 days by 88% (OR = 0.12; 95% CI: 0.10–0.14).

**Conclusion:**

Our study demonstrates that compared to iHD, CRRT is associated with a significantly lower risk of dialysis dependence and progression to ESRD in patients with AKI. CRRT may prevent further kidney injury and promote improved renal recovery.

## Introduction

Renal replacement therapy (RRT) is a critical intervention when conservative medical management of acute kidney injury (AKI) fails to restore kidney function. The decision to initiate RRT is influenced by several indications, such as worsening acidosis, severe fluid overload, and electrolyte abnormalities, such as hyperkalemia [1]. The timing of RRT initiation is a crucial factor that can impact the patient’s outcome, as early intervention may improve prognosis [2]. However, several other variables, including the underlying cause of the AKI, the patient’s hemodynamic stability, and their response to other treatments, also play key roles in determining the need for RRT [3].

Patients who require RRT typically face worse outcomes compared to those who can recover with medical management alone. The choice between iHD and CRRT is often guided by the patient’s hemodynamic status and the specific need for fluid removal [4]. iHD is often preferred for stable patients with less severe fluid overload or those who do not require continuous monitoring. CRRT, on the other hand, is more commonly employed in critically ill patients who are hemodynamically unstable or who require ongoing, continuous fluid and electrolyte management [5]. These patients may also need mechanical or pharmacological circulatory support, which makes the continuous nature of CRRT a better option in these high-risk settings. Patients receiving CRRT typically have more nonrenal comorbidities because of severe hemodynamic instability and fluid imbalances, which increase their overall risk of complications. If these patients still achieve better ESRD outcomes, it’s particularly significant given the complexity of their underlying conditions.

The use of CRRT in AKI has been extensively studied, but its outcomes remain variable. While some research has shown no survival benefit from CRRT compared to other therapies, other studies have suggested that early initiation of CRRT may be associated with reduced mortality rates [6]. For instance, data suggest that starting CRRT early in the course of AKI can lower the degree of mortality, especially in critically ill patients with other complications. As more research emerges on the use of CRRT in critically ill populations, particularly its safety, efficacy, and long-term outcomes, the evidence base continues to evolve, offering more nuanced insights into its role in managing severe AKI [7].

Several studies have explored the relationship between the need for RRT and the long-term prognosis of patients with AKI, particularly the risk of developing dialysis dependence or progressing to end-stage renal disease (ESRD). One such study [8], conducted in 2013, focused on the mortality and long-term outcomes of a cohort of 863 ICU patients who were initiated on CRRT between 2008 and 2011. These patients had varying degrees of AKI and ESRD. The study found that hospital mortality was significantly higher in AKI patients, at 61%, compared to 54% in those with ESRD. The study identified key risk factors for mortality, including older age, a baseline creatinine level greater than 3 at the time of CRRT initiation, lactate levels greater than 4, and the presence of comorbid liver disease. These factors were associated with a higher likelihood of poor outcomes, underscoring the importance of early and precise identification of patients at high risk of deterioration.

Another study, published in 2021 [9], expanded on these findings by examining a larger cohort of 1,135 patients with AKI. This retrospective study assessed mortality rates and the need for CRRT reinstitution or liberation (discontinuation of therapy). The study found that mortality was highest in patients who were liberated from CRRT (62%), followed closely by those who had CRRT reinstituted (59%). Interestingly, the mortality rate was 40%. This study further revealed poor 90-day outcomes for AKI patients treated with CRRT, as a substantial number of patients required long-term dialysis or had irreversible kidney damage. Despite these findings, some data from different points in time have shown that CRRT can offer a benefit in the management of severe AKI, especially in terms of improving short-term survival in hemodynamically unstable patients.

The risk of needing long-term dialysis after an episode of AKI and the progression to ESRD is a subject of ongoing research. Several major trials and studies have focused on identifying predictors of dialysis dependence after AKI, including factors such as the duration of CRRT, the underlying cause of the AKI, and the patient’s overall health status [10]. In general, AKI that is severe or prolonged, particularly when associated with comorbidities, such as diabetes, hypertension, or liver disease, increases the likelihood that the patient may eventually require chronic dialysis [11].

Given the high morbidity and mortality associated with AKI requiring RRT, our study aimed to investigate the real-time risk of dialysis dependence and progression to ESRD in patients with AKI. We conducted a retrospective analysis using the MarketScan database, a large administrative claims database, to identify the incidence and likelihood of requiring HD in patients with AKI. By analyzing these data, we aimed to shed light on the long-term outcomes of AKI patients, particularly the factors that increase the risk of dialysis dependence and ESRD progression within 90 days of discharge. Our analysis seeks to contribute to a better understanding of the prognosis for these patients and potentially identify early interventions that could reduce the long-term burden of dialysis and kidney failure.

## Methods

### Search strategy

This analysis was conducted using data extracted from the MarketScan Commercial Claims and Encounters database (Marketscan, Merative), covering the period from 2005 to 2021. MarketScan is a multi-institutional, de-identified database containing patient data from across the United States without limitations on geography or demographics. The database contains reimbursed claims within the United States from >130 payers for >56 million employees, dependents, and retirees covered annually under private insurance plans. No Medicaid or Medicare data are included. Patients are included for the duration of their enrollment in a given insurance plan, and the database includes inpatient claims, outpatient visits, emergency department visits, and pharmaceutical claims. The database allows for large-scale, longitudinal research studies in various fields of healthcare, including the management of acute kidney injury (AKI).

### Inclusion criteria

We identified all inpatient admissions with an International Classification of Diseases 9^th^ (ICD-9) or 10^th^ (ICD-10) diagnosis code for AKI (ICD-9: 584, 584.4, 584.6-584.9; ICD-10: N17, N17.0-N17.2, N17.8, N17.9) from 2005 to 2021. For patients with multiple admission in this time frame, we used only the first admission. We included adult patients (age ≥18 years old) with continuous enrollment in an insurance plan from 90 days prior to admission to 97 days after discharge from admission. Ninety-seven days was used because we included a 7-day window for outcomes at 90 days after discharge. Patients were also required to have either hemodialysis or CRRT (or both) during the admission. These were determined using Current Procedural Terminology (CPT) codes of 90935 (hemodialysis) or 90945 (CRRT).

We excluded patients with hemodialysis or CRRT within 90 days prior to admission, and those with end stage renal disease (ESRD) or a kidney transplant for the same time frame. ESRD was identified using an ICD-9/10 diagnosis codes of 585.6 and N18.6. Kidney transplant was identified using ICD-9/10 diagnosis codes of V42.0 and Z94.0 (Table 1).

**Table 1.** Patient characteristics stratified by treatment groups.

	Hemodialysis (*N* = 16,189)	CRRT (*N* = 2,116)	Both (*N* = 2,565)	Total (*N* = 20,870)	*p*-value
Age, mean (SD^1^]	51.1 (10.9)	50.1 (12.1)	50.4 (11.3)	50.9 (11.1)	0.003
					
Gender, *n* (%)					<0.001
Male	9801 (60.5%)	1217 (57.5%)	1636 (63.8%)	12654 (60.6%)	
Female	6388 (39.5%)	899 (42.5%)	929 (36.2%)	8216 (39.4%)	
					
Year of hospitalization, *n* (%)					<0.001
2005–2008	4431 (27.4%)	346 (16.4%)	454 (17.7%)	5231 (25.1%)	
2009–2012	5276 (32.6%)	612 (28.9%)	815 (31.8%)	6703 (32.1%)	
2013–2016	3628 (22.4%)	526 (24.9%)	649 (25.3%)	4803 (23.0%)	
2017–2021	2854 (17.6%)	632 (29.9%)	647 (25.2%)	4133 (19.8%)	
					
Length of stay, median (IQR^2^)	13 (8–23)	21 (11–35)	32 (21–50)	16 (9–28)	<0.001

^1^SD: Standard deviation

^2^IQR: Interquartile range.

### Study outcomes

Our primary focus was to investigate the risk of dialysis dependence at discharge and at 90 days post-discharge.

To identify dialysis dependence, we required that a patient had one of the following [1]: an ICD-9/10 code of V45.11 or Z99.2, which denote long-term dialysis dependence, on the day of discharge; or [2] a CPT code indicating hemodialysis or CRRT from the day after discharge until 7 days after discharge; or [3] an ICD-9/10 diagnosis code for ESRD (585.6 or N18.6) on the day of discharge. We used the same definition for dialysis dependence at 90 days. Thus, the window for hemodialysis or CRRT (second criteria above) occurred from 91 to 97 days after discharge. We also evaluated ESRD (the third criteria in the definition above) at any point from discharge to 90 days after discharge.

### Assessment of potential covariates

Demographic information, including gender, age at hospitalization, and year of hospitalization, was extracted from the MarketScan database. Additionally, we calculated the length of hospital stay (LOS) during the index admission.

### Study design and statistical analysis

The primary outcome of the study was to assess the impact of treatment for AKI (hemodialysis and/or CRRT) on outcomes, specifically dialysis dependence at discharge and at 90 days after discharge, and a diagnosis of ESRD at any point within 90 days after discharge.

Patient and admission characteristics were compared among treatment groups using chi-squared tests for categorical variables and Kruskal–Wallis tests for continuous variables. Outcomes were compared among groups using chi-squared tests.

We used logistic regression to examine the relationship between treatments and each outcome, all of which are binary. The regression models included treatment group (hemodialysis, CRRT, or both) during the index admission, age, sex, calendar year, and LOS. For the outcomes at 90 days, we also included dialysis dependence at discharge in the model. For these models, age and calendar year were modeled linearly, which was appropriate based on spline fits and other graphical analyses. LOS was modeled using a restricted cubic spline with 4 degrees of freedom, which allowed for a non-linear association between LOS and outcomes. All other variables were categorical and used reference coding. Odds ratios (ORs) and corresponding 95% confidence intervals (CIs) and *p*-values were reported from these models.

## Results

A total of 20,870 patients were identified with a diagnosis of AKI requiring RRT during an inpatient admission. For the sample, 16,189 received hemodialysis, 2,116 patients received CRRT, and 2565 received both during the index inpatient admission.

### Study characteristics

Baseline characteristics were stratified by the treatment group in Table 1. All variables had statistically significant differences (*p* < 0.05) among treatment groups. However, differences in mean age and sex were small, whereas median LOS differed substantially among groups (medians of 13, 21, and 32 days).

### Outcomes

Dialysis dependence at discharge occurred in 4130 patients (19.8%), dialysis dependence at 90 days occurred in 2892 patients (13.9%), and ESRD within 90 days after discharge occurred in 9213 patients (44.1%). These outcomes stratified by treatment groups are shown in Table 2.

**Table 2.** Estimated ORs from fitted logistic regression models for each outcome.

	Dialysis dependent at discharge		Dialysis dependent at 90 days		ESRD within 90 days	
Parameter	OR (95% CI)	*p*-value	OR (95% CI)	*p*-value	OR (95% CI)	*p*-value
Treatment group						
Hemodialysis (ref)	1		1		1	
CRRT	0.33 (0.28–0.39)	<0.001	0.20 (0.16–0.27)	<0.001	0.12 (0.10–0.14)	<0.001
Both hemodialysis and CRRT	1.41 (1.27–1.57)	<0.001	0.54 (0.45–0.64)	<0.001	0.65 (0.58–0.72)	<0.001
Age, 10-year increase	1.03 (0.99–1.06)	0.10	1.03 (0.99–1.07)	0.174	1.08 (1.05–1.11)	<0.001
Sex						
Male (ref)	1		1		1	
Female	1.01 (0.94–1.08)	0.86	1.13 (1.04–1.23)	<0.001	0.91 (0.86–0.97)	0.005
Length of stay*		<0.001		<0.001		<0.001
28 vs. 9 days	0.80 (0.73–0.88)	<0.001	0.48 (0.44–0.52)	<0.001	0.39 (0.35–0.42)	<0.001
47 vs. 9 days	0.62 (0.55–0.69)	<0.001	0.41 (0.36–0.47)	<0.001	0.32 (0.29–0.35)	<0.001
Year of hospitalization, 5-year increase	1.07 (1.03–1.12)	<0.001	0.99 (0.94–1.04)	0.57	0.91 (0.88–0.94)	<0.001
Dialysis-dependent at discharge	--		4.71 (4.32–5.14)	<0.001	6.82 (6.25–7.43)	<0.001

*Length of stay modeled non-linearly, OR estimates provided for 75^th^ vs. 25^th^ percentile and 90^th^ vs. 25^th^ percentile.

All outcomes differed substantially between groups, with CRRT having a lower percentage of patients with each outcome compared to hemodialysis: 7.5% versus 20.9% for dialysis dependence at discharge, 2.8% versus 16.4% at 90 days, and 9.2% vs. 50.6% for ESRD within 90 days after discharge.

The estimated odds ratios from fitted logistic regression models for each outcome are shown in Tables 3–5. For all three outcomes, patients treated with CRRT had lower odds of each outcome compared to those with hemodialysis. In particular, compared to those patients receiving hemodialysis, patients receiving CRRT had 67% lower odds of being dialysis dependent at discharge (OR = 0.33; 95% CI: 0.28–0.39), had 80% lower odds of being dialysis dependent at 90 days (OR = 0.20; 95% CI: 0.16–0.27), and had 88% lower odds of developing ESRD within 90 days (OR = 0.12; 95% CI: 0.10–0.14). LOS was highly significant in all models and indicated the longer the LOS, had lower the odds of each outcome.

**Figure 1.**
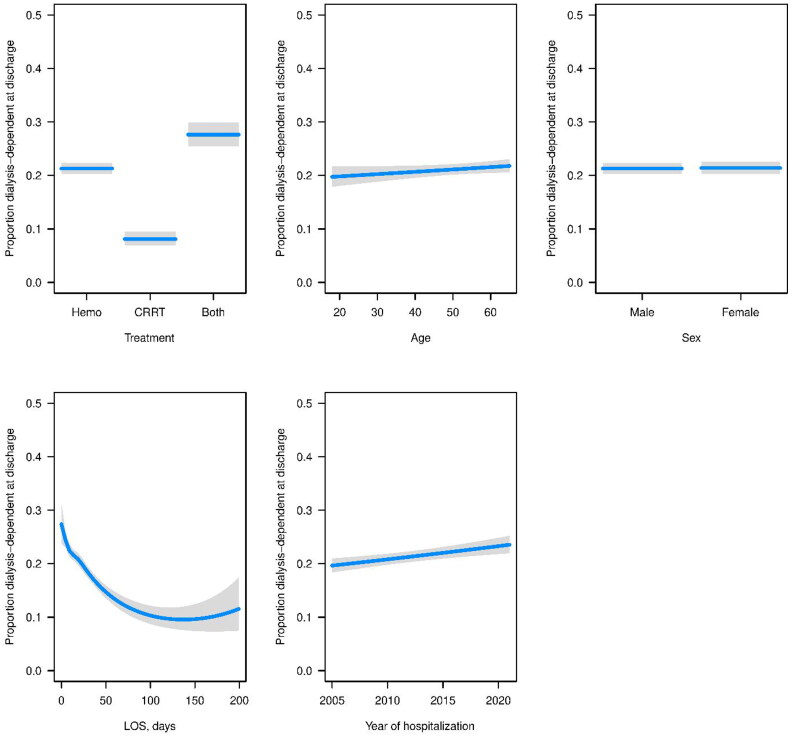
Estimated probabilities (blue lines) and corresponding 95% CIs (gray regions) for each variable from fitted logistic regression model for dialysis-dependence at discharge. For each variable, all other variables in the model were set to the median (continuous variable) or most common response (categorical variable).

**Figure 2.**
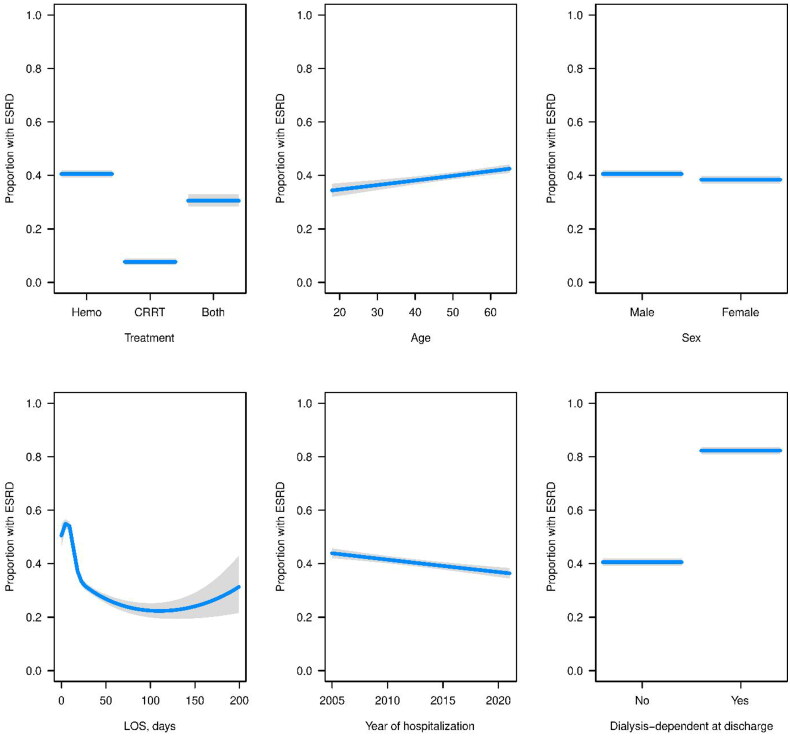
Estimated probabilities (blue lines) and corresponding 95% CIs (gray regions) for each variable from the fitted logistic regression model for dialysis-dependence at 90 days. For each variable, all other variables in the model were set to the median (continuous variable) or the most common response (categorical variable).

**Figure 3.**
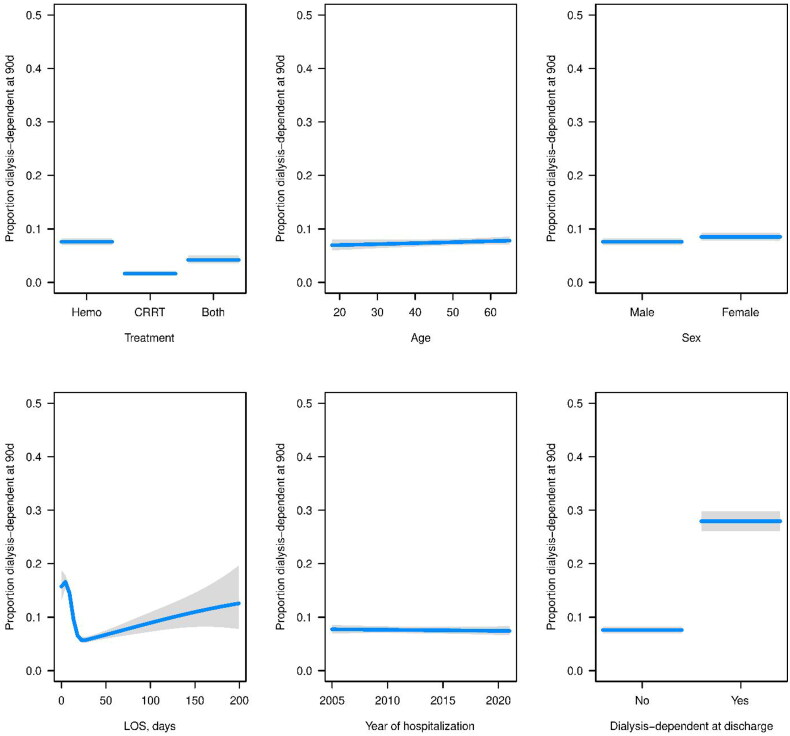
Estimated probabilities (blue lines) and corresponding 95% CIs (gray regions) for each variable from the fitted logistic regression model for ESRD within 90 days. For each variable, all other variables in the model were set to the median (continuous variable) or the most common response (categorical variable).

For dialysis dependence at discharge, other significant variables included treatment with both CRRT and hemodialysis, which had worse odds compared to hemodialysis alone, and year of hospitalization, which indicated that later years had higher odds of dialysis dependence. For dialysis dependence at 90 days, treatment with both hemodialysis and CRRT had significantly lower odds, as did female sex. For ESRD within 90 days, treatment with both hemodialysis and CRRT had significantly lower odds, as did male sex, and later year of hospitalization (Figures 1–3).

### Logistic regression models

The estimated ORs from fitted logistic regression models are given in (Table 2).

## Dialysis dependence at discharge

Results of dialysis dependence at discharge are shown in Figures 1.

### Dialysis dependence at 90 days

Estimated probabilities and corresponding 95% CIs for each variable from the fitted logistic regression model are shown in Figures 2.

### ESRD within 90 days

Estimated probabilities corresponding 95% CIs (gray regions) for each variable from the fitted logistic regression model for ESRD are given in Figures 3.

## Discussion

Our study provides substantial evidence that CRRT is associated with improved outcomes in critically ill patients with AKI, particularly in terms of reduced dialysis dependence at both discharge and 90 days, as well as a lower incidence of ESRD. These findings are important as they suggest that CRRT may offer distinct advantages over iHD in this patient population, especially for those with more severe or complex forms of AKI who require RRT.

A key insight from our study is the consistent association between CRRT and a decreased risk of dialysis dependence and progression to ESRD. The continuous, gradual nature of CRRT, which operates over 24 h, provides a more controlled and stable removal of solutes and fluids [12]. This stands in contrast to the episodic nature of iHD, where large volumes of fluids are removed rapidly during a session. These rapid shifts in fluid and electrolytes in iHD can induce hemodynamic instability, potentially aggravating renal injury and leading to further damage to the kidneys, which in turn increases the risk of sustained dialysis dependence and progression to ESRD [13]. The less aggressive, continuous nature of CRRT may therefore protect the kidneys from additional injury and promote recovery, particularly in patients who are critically ill and are more vulnerable to the adverse effects of rapid volume shifts.

Moreover, the hemodynamic stability offered by CRRT likely plays a critical role in its association with better outcomes. CRRT is better tolerated in patients who are hemodynamically unstable, which is common in those with severe AKI [14]. The continuous slow fluid removal avoids the variability in blood pressure and volume status that may be seen with iHD, providing a gentler approach that supports more stable cardiovascular function. This can be crucial in preventing the cycle of worsening kidney injury and subsequent complications such as fluid overload, which can further exacerbate AKI and increase the risk of progression to ESRD. All of these factors make CRRT the preferred choice over IHD in AKI for patients with hemodynamic instability, severe fluid overload, multi-organ dysfunction, significant electrolyte imbalances, and a high risk of dialysis dependence, as it ensures gradual, continuous renal support with better hemodynamic stability.

However, it is crucial to acknowledge that patients who receive CRRT are often more critically ill than those who receive iHD. These patients may have a higher baseline risk of poor outcomes due to the severity of their illness. Therefore, while our data suggest that CRRT is associated with better outcomes, the inherent illness severity of CRRT patients could confound the results. It is important to consider that the lower dialysis dependence and ESRD progression observed in CRRT patients could reflect the overall benefit of CRRT as a more supportive therapy, but it is also possible that patients receiving iHD might have had a different risk profile that influenced their outcomes independently of the dialysis modality. These nuances underline the complexity of interpreting results from observational studies and the need for further controlled trials to establish more definitive causal relationships.

### Current literature

Our findings contribute to the broader discussion on CRRT versus IHD in AKI management, aligning with previous studies suggesting that CRRT may help preserve kidney function and reduce long-term dialysis dependence, particularly in hemodynamically unstable patients [15]. The continuous, gradual nature of CRRT minimizes rapid fluid and solute shifts, which are common in IHD and can precipitate hemodynamic instability, potentially exacerbating renal injury. Prior research has similarly highlighted the benefits of CRRT in maintaining hemodynamic stability, supporting its role in critically ill patients who are vulnerable to fluctuations in blood pressure and volume status [16].

However, not all studies have found a clear advantage of CRRT over IHD in terms of long-term renal recovery(ref). Some investigations suggest that the timing of CRRT initiation plays a critical role, with early initiation not always translating to better outcomes compared to a more conservative approach [17]. These differences may be influenced by variations in patient populations, institutional practices, and treatment protocols. While our study reinforces the association between CRRT and reduced dialysis dependence, the inherent severity of illness in CRRT patients introduces potential selection bias, making it challenging to determine whether these benefits are directly attributable to CRRT or other clinical factors.

### Clinical relevance

Identifying factors associated with dialysis dependence after an acute kidney injury event is of the essence, as these factors impact patient outcomes and the utilization of healthcare resources. On average, CRRT use costs $ 870 USD per day, and the risk of needing dialysis after discharge remains more associated with costs [18]. Longer stay duration can be attributed if these measures failing. Studies have established that the use of CRRT can be associated with variable outcomes. Our study suggests that the use of CRRT can lead to reduced dialysis dependence and ESRD. Knowledge of this can help physicians act immediately on AKI in hopes it would reducing dialysis needs.

### Strengths and limitations

#### Strengths

The major strength of our study is its large sample size, drawn from a real-world dataset, which significantly enhances the generalizability of the findings. The diverse patient population captured through the MarketScan database provides a broad spectrum of demographics, comorbidities, and clinical characteristics, making the findings applicable to a wide range of clinical settings. Additionally, the extensive follow-up period (up to 90 days) allows for a robust assessment of both short-term and longer-term outcomes, including the critical issue of progression to ESRD. The richness of the data on treatment types, patient characteristics, and outcomes adds robustness to our analysis and lends credibility to the observed associations.

#### Limitations

Several limitations must be acknowledged. First, as an observational study, causality cannot be definitively established. While we observed strong associations between CRRT and improved outcomes, we cannot conclusively prove that CRRT directly causes these benefits. There are numerous unmeasured confounding factors, such as the specific timing of intervention, comorbidities, disease severity, and patient preferences, that could affect both the choice of dialysis modality and patient outcomes.

Furthermore, the use of EHR databases like MarketScan introduces the possibility of data inaccuracies, such as misclassification or underreporting of diagnostic codes, procedures, or medications. These inaccuracies could lead to biased conclusions if they differ systematically between the groups receiving CRRT and iHD. Moreover, due to the nature of data in MarketScan, the study did not include patients whose only insurance was Medicaid or Medicare potentially reducing generalizability to populations covered by Medicaid or Medicare, particularly older adults and individuals with lower socioeconomic status. Additionally, the observational nature of the study raises concerns about selection bias, as patients who receive CRRT may differ from those who receive iHD in ways that are not fully captured in the data. For instance, patients requiring CRRT may have had more severe or complex clinical conditions that influence their risk of poor outcomes independently of the dialysis modality used. The use of data from the MarketScan database presents inherent limitations, including the potential for misclassification and underreporting of diagnostic codes and procedures. These data quality concerns may introduce bias, which could impact the accuracy and interpretation of our findings. Another important limitation is the lack of access to clinical variables that may influence kidney recovery, such as baseline kidney function, AKI etiology, hemodynamic status, ICU admission, and comorbidities. These were not available in the MarketScan claims dataset, limiting our ability to adjust for disease severity. Including comorbidities would have required a longer pre-admission enrollment period (6–12 months), while our study used a 90-day look-back. Similarly, AKI severity could not be assessed, as diagnosis codes lack the clinical detail to capture it accurately. Lastly, the 9, 28, and 47-day time points for length of stay in our regression model reflect the 1st quartile, median, and 3rd quartile of our data and were used to illustrate non-linear effects, not as arbitrary cutoffs.

Another limitation is the lack of randomization in our study, inherent to observational studies. Although the large sample size and real-world data offer strong evidence, the absence of random assignment means that patients who receive CRRT may differ from those who receive iHD in terms of other clinical factors, such as overall treatment strategy or medical interventions. This introduces the risk of unmeasured confounding, where differences between the two groups, such as the choice of therapy, could influence the outcomes observed.

We acknowledge the potential for selection bias, as patients receiving CRRT may have more severe underlying conditions compared to those receiving iHD. This difference in baseline disease severity and comorbidities could influence our findings, and we recognize that the lack of adjustment for these factors is a limitation of our study.

## Conclusion

In conclusion, our study suggests that CRRT is associated with better outcomes in terms of reduced dialysis dependence and a lower risk of ESRD in critically ill AKI patients compared to intermittent hemodialysis. The continuous, gradual removal of fluids and solutes in CRRT likely contributes to its more hemodynamically stable and kidney-protective nature, which may prevent further renal damage and support recovery in severely ill patients. However, due to the observational design of the study, these findings should be interpreted with caution, and further randomized controlled trials, which compare the severity of disease indices, are needed to confirm the causal relationship between CRRT and improved patient outcomes. Ultimately, CRRT remains a promising option for renal replacement therapy in critically ill AKI patients, but clinical decision-making should be individualized based on patient severity, hemodynamic stability, and other clinical factors.

## Data Availability

The data used in this study are available upon reasonable request. Researchers interested in obtaining the data may contact the corresponding author for further details on the access process.
